# Development and Validation of a Diabetes Risk Prediction Model With Individualized Preventive Intervention Effects

**DOI:** 10.1210/clinem/dgaf250

**Published:** 2025-04-23

**Authors:** Byron Jaeger, Ramon Casanova, Yitbarek Demesie, Jeanette Stafford, Brian Wells, Michael Patrick Bancks

**Affiliations:** Department of Epidemiology & Prevention, Wake Forest University School of Medicine, Winston-Salem, NC 27157, USA; Department of Epidemiology & Prevention, Wake Forest University School of Medicine, Winston-Salem, NC 27157, USA; Department of Epidemiology & Prevention, Wake Forest University School of Medicine, Winston-Salem, NC 27157, USA; Department of Epidemiology & Prevention, Wake Forest University School of Medicine, Winston-Salem, NC 27157, USA; Department of Epidemiology & Prevention, Wake Forest University School of Medicine, Winston-Salem, NC 27157, USA; Department of Epidemiology & Prevention, Wake Forest University School of Medicine, Winston-Salem, NC 27157, USA

**Keywords:** type 2 diabetes prevention, intensive lifestyle intervention, metformin therapy, risk prediction, precision medicine

## Abstract

**Objective:**

Type 2 diabetes risk prediction models lack the option to predict risk conditional on initiating different preventive interventions. Our objective was to develop and validate a diabetes risk prediction model with individualized preventive intervention effects among racially diverse populations.

**Methods:**

The derivation cohort included participants in the Diabetes Prevention Program (DPP) trial randomized to placebo, metformin, or intensive lifestyle intervention (n = 2640). A risk prediction model for incident diabetes was developed using Cox proportional hazards regression using clinically available predictors: sex, glycated hemoglobin, fasting plasma glucose (FPG), body mass index (BMI), triglycerides, and intervention. To create individualized intervention effects, pairwise interactions between intervention and age, FPG, and BMI were included. The discrimination, calibration, and net benefit of the model's 3-year predictions for incident diabetes were internally validated within the DPP and externally validated among participants with prediabetes in the Multi-Ethnic Study of Atherosclerosis (MESA; n = 2104).

**Results:**

In DPP and MESA, mean (SD) age was 51 years (11) and 64 (10), and 67% and 50% of participants were women, respectively. The mean C-statistic was 0.71 [95% confidence interval (CI): 0.68, 0.74] in DPP and 0.86 (95% CI: 0.83, 0.88) in MESA. The optimal preventive intervention (lowest 3-year risk) was lifestyle for 86% and 97% of DPP and MESA participants, respectively, and metformin for the remaining. Model performance was similar across race/ethnicity groups.

**Conclusion:**

This is the first study to develop and validate a diabetes risk prediction model with individualized preventive intervention effects that may improve clinical decision-making and diabetes prevention.

High variation in who will progress from prediabetes to develop type 2 diabetes mellitus poses challenges for clinical decisions related to preventive interventions. The Diabetes Prevention Program (DPP) trial was designed to assess the prevention of diabetes from randomization to intensive lifestyle intervention or metformin therapy compared to placebo (1). Compared to placebo, diabetes risk was 58% lower for the lifestyle arm and 31% lower for the metformin arm over 3 years (2). Not everyone in each intervention arm benefited equally; lifestyle was more effective for older adults, and metformin was more effective with higher baseline glucose and body mass index (BMI). Diabetes risk prediction models developed in the DPP are restricted to include only 1 of the active intervention arms (metformin) or do not include an intervention arm as a model risk predictor and do not enable estimation of an individualized intervention effect and predicted risk (3, 4). Other diabetes risk prediction models in US populations are based on observational study data and cannot quantify the difference in diabetes risk across preventive interventions (5-11). For diabetes prevention, lifestyle modification is the recommended initial strategy with the use of metformin for prevention to be considered for especially high-risk individuals (12). However, no tool exists to allow clinicians and patients the ability to compute and compare predicted risk for diabetes following initiation of first-line preventive interventions vs not starting an intervention. There is a clinical need for diabetes risk prediction models capable of informing individuals how initiation of a preventive intervention may impact their predicted risk of developing type 2 diabetes and identify which intervention approach would be optimal for diabetes prevention for that individual's clinical profile (12, 13). The objective of this study was to develop and validate a model for diabetes risk prediction that incorporates expected benefits for multiple first-line diabetes prevention strategies and includes individualized intervention effects.

## Research Design and Methods

This study was reviewed by the institutional review board of Wake Forest University School of Medicine and approved for exempt status (Exempt Protocol: IRB00091104). Institutional review board approval was obtained for protocols for the Diabetes Prevention Program (DPP) and Multi-Ethnic Study of Atherosclerosis (MESA), and participants in each study provided written informed consent.

### Study Populations and Methods

#### DPP

The derivation sample included data from the DPP randomized clinical trial. Study design, methods, and clinical exam procedures have been reported in detail (1, 14). From 1996 to 1999, 3819 individuals ages 25 to 85 years were enrolled at 27 clinical centers across the United States (1). A 4-step process consisted of consent, screening, and recruitment of participants who were at high risk for type 2 diabetes based on weight and glucose status. This included an initial fasting plasma glucose (FPG) level assessment and subsequent 75-gram oral glucose tolerance test (OGTT). Major eligibility criteria included age ≥25 years, BMI ≥24 kilograms per meter squared (kg/m^2^; ≥22 kg/m^2^ for Asian individuals), FPG of 95 to 125 mg/dL (5.3-6.9 mmol/L, altered from 95-140 mg/dL in 1997), and 2-hour OGTT glucose of 140 to 199 mg/dL (7.8-11.0 mmol/L). Major exclusions included occurrence in the prior 6 months of myocardial infarction, symptoms of coronary heart disease, serious illness, or use of medications known to impair glucose tolerance (1). Of those enrolled, 3665 (66% women) gave consent for their deidentified data to be shared with the public. Race/ethnicity was self-reported based on the 1990 census questionnaire and collapsed into 4 categories for the public use data: White (58%), African American (20%), Hispanic (17%), and all other (5%). Of the public use data, “all other” is predominantly individuals who report Asian race/ethnicity.

DPP participants were randomized (stratified by clinical site) to 1 of 4 arms, described previously: intensive lifestyle intervention, standard care plus metformin, troglitazone, or standard care plus placebo tablet (1, 14). Troglitazone was discontinued in 1998 due to concerns of liver toxicity, and participants for this group were not included for analysis (1). Briefly, the protocol for the 3 active groups was as follows. The intensive lifestyle intervention was designed to achieve and maintain 7% weight loss through a healthy, low-calorie and low-fat diet; maintaining moderate-intensity physical activity of ≥150 minutes weekly; and a 16-session behavioral change curriculum over 24 weeks designed to help set and achieve dietary and physical activity goals (monthly afterward). The metformin regimen was titration to 850 mg twice daily (or a manageable dose) with standard lifestyle recommendations and an annual individual lifestyle session with a case manager. The placebo was 1 tablet daily with standard lifestyle recommendations and an annual individual lifestyle session with a case manager.

#### MESA

The external validation sample included participants with prediabetes from the MESA observational cohort. In 2000-2002, 6814 participants (53% women) were recruited at 6 field centers located in Baltimore, Maryland; Chicago, Illinois; Forsyth County, North Carolina; Los Angeles, California; New York, New York; and Saint Paul, Minnesota (15). Participants were ages 45 to 84 years and free of clinical cardiovascular disease at enrollment from 4 racial/ethnic groups (self-identified): non-Hispanic white (38%), African American (28%), Hispanic (22%), and Chinese American (12%). MESA has completed 5 follow-up exams (2002-2004, 2004-2005, 2005-2007, 2010-2011, 2016-2018), all with standardized collection of demographic, socioeconomic, behavioral, clinical, and vascular imaging markers and current medications information. Normal FPG (FPG < 100 mg/dL, <5.6 mmol/L), prediabetes (FPG 100-125 mg/dL, 5.6-6.9 mmol/L), and diabetes (FPG ≥ 126 mg/dL, ≥7.0 mmol/L or use of diabetes medications) status were determined at each MESA exam. At MESA exam 2, glycated hemoglobin (HbA1c) was assessed and used to supplement these classifications. OGTT was not performed in MESA.

### Study Population

DPP participants randomized to the troglitazone arm (n = 584) and who met the current American Diabetes Association criteria of diabetes via FPG or HbA1c (n = 441) at baseline were excluded. The DPP sample (n = 2640) included individuals who met the definition of prediabetes via both fasting and 2-hour post-OGTT glucose. Participants from MESA were included if they had prediabetes identified at exam 1 or exam 2 (if not identified at exam 1). The exam at which prediabetes was first identified was considered the baseline for the risk prediction analysis. Individuals with normal glucose status and diabetes at MESA exams 1 and 2 were excluded from the validation sample. This resulted in an analytic sample for MESA of n = 2104.

### Clinical Risk Predictors

Variables considered for inclusion in the development of the risk prediction model were chosen based on a balance of availability for collection in a clinical or exam setting, biological function, known risk prediction of or causal for developing type 2 diabetes, and DPP intervention response effects (2-10). Based on this balance of criteria, a total of 7 variables were selected a priori to be included in the risk prediction model: FPG HbA1c, BMI, triglycerides, age, sex, and DPP randomization arm.

### Primary Outcome: Diabetes Ascertainment

The primary outcome was incident diabetes (assumed to primarily be type 2 diabetes) until the end of follow-up or last exam attended. In the DPP, diagnosis of type 2 diabetes was ascertained and defined by semiannual measurement of FPG ≥ 126 mg/dL (≥7.0 mmol/L) and annual post-75-gram OGTT 2-hour glucose ≥200 mg/dL (≥11.1 mmol/L) (1). In MESA, diabetes was defined at each study exam as new use of insulin or oral hypoglycemic medications or FPG ≥126 mg/dL (≥7.0 mmol/L) (16, 17).

### Statistical Analysis

Participant characteristics were summarized overall and among DPP and MESA participants separately. Between cohorts, continuous variables were harmonized to similar units and categorical variables to similar categorical definitions. Continuous variables were summarized using mean (SD) or median (25th, 75th percentile), and categorical variables were summarized using percentage. Missing data for risk prediction model variables were imputed by aggregating the value from 5 of the observation's nearest neighbors according to Gower's distance (18).

Cox regression was applied to develop a risk prediction model for incident type 2 diabetes among DPP participants. The model included main effects for FPG, HbA1c, BMI, lipids, age, sex, and DPP randomization arm, with additional pairwise interactions between randomization arm and age, FPG, and BMI. To evaluate the utility of individualizing preventive intervention effects on risk predictions, a standard “nonindividualized” model was developed using the same main effects as the individualized model but without the pairwise interactions.

### Model Estimation and Evaluation of Performance

Both model specifications were internally validated using 10-fold cross-validation among DPP participants and were also fitted to the entire DPP sample. Risk predictions at 3 years after baseline from these finalized models were externally validated among MESA participants. For both internal and external evaluation, predictions were evaluated in terms of discrimination, calibration, net reclassification index, net benefit, and multiple measures of fairness. Discrimination was estimated using the area under the receiver operating characteristic curve concordance (C-) statistic, which measures the probability of assigning higher risk to a case vs a noncase (19). Calibration was evaluated in terms of agreement between average observed risk and deciles of predicted risk. The net reclassification index (NRI) is based on positive and negative net reclassification, which are both scored based on how well a new model “reclassifies” cases and noncases relative to a standard model. In our analyses, the individualized model played the role of a new model while the nonindividualized model was the standard. Net benefit is based on the weighted difference in the true vs false positive rate of a prediction model at a given decision threshold, ie,


netbenefit=truepositiverate−(falsepositiverate*exchangerate),

where the exchange rate is the odds of the threshold probability used for clinical decision-making (20). Net benefit can be interpreted as the number of true-positive cases identified per 100 patients screened (21). Fairness was measured in terms of equal opportunity and equal odds, with each metric based on equity in subgroups defined by race and sex (22, 23).

The final prediction model with individualized preventive intervention effects was used to calculate counterfactual risk (ie, “what if” scenarios) for participants in DPP and MESA to assess 3-year predicted risk of incident diabetes conditional on the participant initiating lifestyle, metformin, or no intervention. The predicted optimal prevention strategy for an individual was determined as the intervention scenario with the lowest 3-year predicted risk. We computed the number needed to treat to prevent 1 incident case of diabetes over 3-years follow-up using 3 policies: (1) treat everyone with lifestyle intervention, (2) treat everyone with metformin therapy, and (3) treat according to the predicted optimal prevention strategy (eg, metformin if predicted risk with metformin is lowest). This model was also deployed in a freely available web application that calculates and summarizes an individual's estimated risk for diabetes under each intervention scenario, and this diabetes risk calculator is published at https://diabetesriskcalculator.phs.wakehealth.edu. Analyses were conducted using SAS version 9.4 (SAS Institute, Cary, NC, USA) and R version 4.4.0 (The R Foundation), and statistical tests were 2-sided with α = .05.

## Results

Baseline characteristics including demographics and clinical risk factors for type 2 diabetes of the DPP (derivation) and MESA (validation) analytic samples are presented in Table 1. The DPP sample mean ± SD age was 51 years ± 11 and was 67% female, 62% non-Hispanic White, 16% non-Hispanic Black, 17% Hispanic, and 5% other race/ethnicity. Over three-quarters of DPP participants had greater than a high school education. Compared to DPP, the MESA sample was older and had a greater proportion of men and non-Hispanic Black, Hispanic, and Chinese race and lower educational attainment. Mean ± SD BMI was 34 kg/m^2^ ± 7 in DPP and 30 kg/m^2^ ± 6 in MESA. DPP participants had higher mean values for blood glucose, insulin resistance, and β-cell function than MESA participants. Characteristics of DPP and MESA participants who were excluded from analysis are shown in Supplementary Table S1 (24).

**Table 1.** Baseline characteristics of the DPP (derivation) and MESA (external validation) participants included in the current analysis

Characteristic*^a^*	DPP, n = 2640	MESA, n = 2104
Age, years	51 (11)	64 (10)
Sex (%)		
Male	864 (33)	1058 (50)
Female	1776 (67)	1046 (50)
Race/ethnicity (%)		
Non-Hispanic White	1626 (62)	682 (32)
Non-Hispanic Black	423 (16)	558 (27)
Hispanic	448 (17)	510 (24)
Other*^b^*	143 (5.4)	354 (17)
Educational attainment (%)		
<High school	175 (7)	196 (23)
High school graduate	467 (18)	168 (20)
Some college or college graduate	1998 (76)	496 (58)
Fasting glucose, mg/dL	106 (7)	101 (10)
Glycated hemoglobin, %	5.78 (0.40)	5.75 (0.30)
HOMA-Insulin Resistance	6.0 (4.2, 8.6)	2.3 (1.6, 3.30
HOMA-β cell function	199 (136, 272)	107 (77, 152)
Body mass index, kg/m^2^	34 (7)	30 (6)
Triglycerides, mg/dL	144 (101, 205)	118 (84, 167)
Low-density lipoprotein cholesterol, mg/dL	107 (27)	116 (32)
High-density lipoprotein cholesterol, mg/dL	46 (12)	50 (14)

Abbreviations: DPP, Diabetes Prevention Program; HOMA, Homeostatic Model Assessment; MESA, Multi-Ethnic Study of Atherosclerosis.

^*a*^Values are count (column percentage), mean (SD), or median (25th, 75th percentile).

^*b*^Other race/ethnicity is predominantly self-reported Asian for the DPP public use data and Chinese for the MESA data.

At 3 years of follow-up, there were 386 incident cases of type 2 diabetes [17% cumulative incidence; 95% confidence interval (CI): 16, 19] in DPP and 202 incident cases in MESA (9.8% cumulative incidence; 95% CI: 8.5, 11).

### Model Comparisons

**Figure 1.**
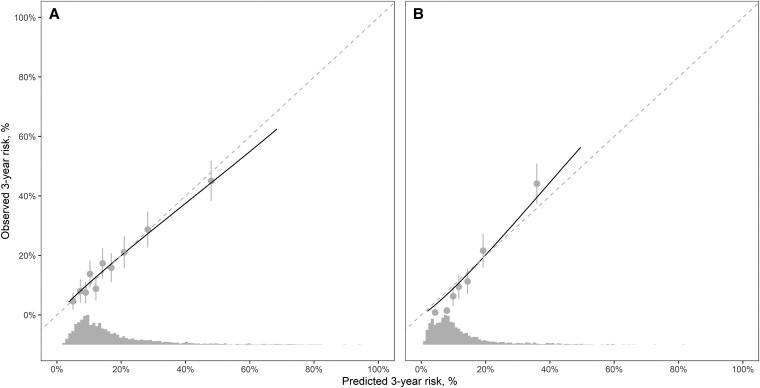
Calibration with histogram of the individualized intervention effect risk prediction model in the diabetes prevention program (panel A, internal validation) and Multi-Ethnic Study of Atherosclerosis (panel B, external validation).

Among DPP participants, the model with individualized preventive intervention effects obtained a C-statistic of 70.7 while the nonindividualized model had a C-statistic of 69.8 (Supplementary Table S2) (24). The individualized model obtained an overall NRI of 0.025 (95% CI −0.0041, 0.053) compared to the nonindividualized model among DPP participants, with improvement in risk reclassification primarily in downclassification of risk (ie, fewer false positives). Among MESA participants, the models obtained better C-statistics than in DPP, and the NRI of the individualized model was 0.0060 (95% CI: −0.0052, 0.021). Model performance metrics were similar or better for the model with individualized preventive intervention effects compared to the nonindividualized model among subgroups for race/ethnicity and sex among DPP participants, and performance metrics were similar across models among MESA participants. The individualized model did not show signs of miscalibration in internal validation among DPP participants, but it did under- and overpredict risk for MESA participants with low and high observed risk, respectively (Fig. 1). Supplementary Figs. S1 to S6 display calibration plots among subgroups for race/ethnicity and sex for DPP and MESA participants, respectively (24). Supplementary Tables S3 to S5 present reclassification matrices comparing the individualized preventive intervention effects model with the standard nonindividualized model by cohort, sex, and race/ethnicity group, respectively (24). Reclassification was more prevalent for DPP vs MESA; women vs men; and non-Hispanic White vs non-Hispanic Black, Hispanic, and other race/ethnicity groups, respectively (24). Using the model with individualized preventive intervention effects among DPP and MESA at a 20% risk threshold was estimated to result in a net benefit of 4 and 3 true positive cases identified per 100 screened, respectively (Supplementary Fig. S7) (24).

### Risk Estimates

The associations between predictors and incident diabetes for the model with individualized preventive intervention effects are reported in Table 2 with instructions to compute predicted risk in Supplementary Table S6 (24). For 86% of DPP participants and 97% of MESA participants, assignment to the intensive lifestyle intervention was optimal for diabetes prevention and resulted in the lowest 3-year mean predicted risk for diabetes (Table 3). For the remaining participants in each respective sample, assignment to metformin was the optimal preventive intervention strategy. For those in DPP where lifestyle was the optimal preventive intervention, the mean 3-year risk for diabetes was 10.0% if assigned to lifestyle, 17.0% if assigned to metformin, and 22.0% if assigned to placebo. For those in DPP where metformin was the optimal preventive intervention, the mean 3-year risk for diabetes was 20.0% if assigned to lifestyle, 15.0% if assigned to metformin, and 27.0% if assigned to placebo. Supplementary Table S7 presents participant characteristics at baseline stratified by the diabetes prevention intervention strategy with the optimal benefit (ie, lowest 3-year risk) (24). Characteristics with greater predicted benefit from lifestyle intervention included older age and lower baseline fasting glucose, while greater benefit from metformin was predicted for individuals with higher BMI (24). This is also illustrated in Supplementary Fig. S8 for 2 hypothetical individuals using the online diabetes risk calculator (24). The number needed to treat to prevent 1 incident case of diabetes was 7.8 when using the intervention approach supported by the new risk prediction model, 8.2 when everyone receives the lifestyle intervention, and 13.1 when everyone receives metformin therapy. Supplementary Fig. S9 displays individualized intervention effect 3-year predicted risk if assigned to each of the interventions (counterfactual risk, “what if” scenario) according to low to high rank order of predicted risk for each respective intervention arm (24). Along the spectrum of predicted risk when assigned to placebo, respective individual predicted risk estimates when assigned to metformin or lifestyle were consistently lower. Along the spectrum of predicted risk when assigned to lifestyle, respective individual predicted risk estimates when assigned to placebo or metformin were consistently higher. Median and interquartile range of individualized 3-year predicted risk for diabetes under each intervention by race/ethnicity and sex are presented in Supplementary Fig. S10 (24). Patterns were similar across demographic groups, with the median risk highest for placebo, then metformin, and lowest for lifestyle.

**Table 2.** Summary of and instructions for using the individualized intervention effect risk prediction model for type 2 diabetes

Variable*^a^*	Hazard ratio (95% CI)*^b^*
Glycated hemoglobin	1.21 (1.09, 1.34)
Triglycerides	1.24 (1.15, 1.35)
Age, per 10.6 years, conditional on treatment	
Lifestyle	0.95 (0.78, 1.16)
Metformin	1.02 (0.86, 1.23)
Placebo	0.97 (0.84, 1.13)
Body mass index, per 6.6 kg/m^2^, conditional on treatment	
Lifestyle	1.37 (1.16, 1.60)
Metformin	0.89 (0.74, 1.06)
Placebo	1.04 (0.91, 1.19)
Fasting glucose, per 6.7 mg/dL, conditional on treatment	
Lifestyle	1.47 (1.23, 1.76)
Metformin	1.53 (1.31, 1.78)
Placebo	1.90 (1.67, 2.17)
Sex	
Female	1.16 (0.95, 1.43)
Male	1.00 (reference)

Abbreviations: CI, confidence interval.

^*a*^Predictor variables included in the table were selected a priori based on clinical availability and known associations with type 2 diabetes risk.

^*b*^Hazard ratios are adjusted for all variables listed in the table. Hazard ratios for continuous variables correspond to a 1 SD change in the variable.

**Table 3.** Optimal preventive intervention and mean 3-year predicted risk (counterfactual risk) for type 2 diabetes from an individualized intervention effect risk prediction model in the Diabetes Prevention Program (derivation) and Multi-Ethnic Study of Atherosclerosis (validation)

Optimal intervention*^a^*	n (% sample)	Mean (SD) 3-year predicted risk, % (counterfactual risk)		
		Lifestyle	Metformin	Placebo
Diabetes Prevention Program (derivation)				
Lifestyle	2267 (86)	10 (7)	17 (9)	22 (16)
Metformin	373 (14)	21 (12)	15 (8)	27 (17)
Multi-Ethnic Study of Atherosclerosis (validation)				
Lifestyle	2035 (97)	7 (5)	14 (10)	15 (15)
Metformin	69 (3)	16 (11)	13 (9)	23 (20)

^*a*^Optimal intervention for an individual is the intervention arm with the lowest 3-year predicted risk for diabetes for each respective individual in the Diabetes Prevention Program. Placebo is never the optimal intervention to prevent diabetes for any individual.

## Discussion

This study used data from a large, randomized trial for type 2 diabetes prevention with multiple intervention arms to develop an individualized intervention effect risk prediction model and equation for developing diabetes over 3 years. This risk prediction model provides an important advancement in diabetes risk prediction and prevention. No current diabetes risk prediction tool can calculate and summarize an individual's estimated risk for developing diabetes after starting a preventive intervention. This model includes 2 unique preventive interventions, intensive lifestyle intervention and metformin therapy; incorporates the differential preventive effects of each intervention; and estimates an individual's risk for diabetes should they either start preventive intervention or not start an intervention (placebo). Thus, it provides an estimate of diabetes risk for each intervention effect that is sensitive to an individual's clinical risk profile (ie, precision preventive medicine) rather than subtracting the mean risk reduction effect of each intervention group from placebo risk. This precision prevention was demonstrated with the model-recommended intervention strategy having the lowest number needed to treat to prevent 1 case of diabetes compared to using lifestyle or metformin for all.

The risk predictor variables included in the model were 6 clinical and biological characteristics and the intervention arm. Race was not included as a model predictor variable because race is not a biological trait. Both analytic samples that contributed to the derivation and validation of the model included racially and ethnically diverse study populations. Across race/ethnicity, the area under the receiver-operator characteristic curve and index of prediction accuracy metrics of model performance were similar. These results support the validity of a race-free diabetes risk prediction model and use of this model among individuals of non-Hispanic White and Black, Hispanic, and Chinese descent. Data on genetic ancestry or genetic risk for diabetes were not included in the publicly available data for DPP, would not be available in a clinical setting, and were not included in the model.

Multiple diabetes risk prediction models, some using DPP data, already exist for US populations, and some of these models have better internal discrimination than the model presented here (3-11). Although the C-statistic for incident diabetes for the model presented here was low overall in the derivation cohort, it was exceptionally high in the validation cohort. Prior models using the DPP trial data show absolute risk reduction ranging from 0% to over 20% across quartiles of estimated type 2 diabetes risk at baseline (3, 4). These prior models are limited due to a lack of external validation, the use of race as a predictor variable, restricting risk prediction to only the metformin arm, and not directly modeling effects of the intervention arms or the heterogeneity by individual clinical factors (3, 4). The existing risk prediction models using observational cohort data can only quantify risk for diabetes under a placebo scenario and lack potential for inference from empirical metformin and lifestyle intervention effects (5-11).

The inclusion of the intervention arm and interaction terms between the intervention arm with weight and fasting glucose are critically important. These parameters can help people understand their risk for developing diabetes on the current standard of care and, if they started either lifestyle or metformin, the current recommended first-line treatments for type 2 diabetes (25). However, not all lifestyle intervention trials support evidence of heterogeneity in diabetes prevention by clinical risk factors and the use of these factors to target interventions (26). An important advantage of this new parsimonious diabetes risk prediction model is that it can be implemented using data available in an electronic health record and clinical setting. This risk prediction equation and accompanying online risk calculator can help inform precision medicine-based efforts for diabetes prevention and guide clinician-patient discussions on patient-specific expected benefit by providing a comparison of diabetes risk across potential intervention strategies. For most people with prediabetes, the intensive lifestyle intervention will be optimal and provide individuals with the lowest risk for developing diabetes over the next 3 years. This was especially evident in MESA, which had a population that was older and had lower BMI than in DPP, which are characteristics shown to strengthen the preventive effect of lifestyle in the original trial (2). For a small subset of individuals, metformin therapy might provide the lowest 3-year risk of diabetes. Where metformin is the optimal intervention (lowest 3-year risk), individuals should accompany metformin therapy with lifestyle modification of diet and physical activity for weight loss, and this is supported by current guidelines (12). Metformin therapy in combination with intensive lifestyle intervention was not assessed in DPP, and this remains a clinical gap in overall and individualized diabetes prevention.

Several limitations merit consideration when interpreting these results. The study populations used to develop and validate the risk prediction model were restricted to individuals with prediabetes. The DPP was a population at high risk for diabetes due to specific pathophysiologic processes, impaired fasting glucose, impaired glucose tolerance, and overweight/obesity. However, the MESA sample was inclusive of broader pathophysiologic processes for prediabetes, and model performance was high in this cohort. Model inference and application to individuals with normal glucose is not advised, though 3-year absolute risk for type 2 diabetes among these individuals is likely very low. Validation was performed in an observational cohort where no study-based preventive interventions were implemented, which may have contributed to the underestimation of predicted risk at low observed risk and overestimation at high observed risk. MESA participants may have freely participated in diabetes prevention programs, but this information was not collected. The preventive interventions, metformin and intensive lifestyle modification, were assumed to follow the protocol employed in the DPP trial, and modeling does not account for crossover or nonadherence to intervention. Newer pharmacotherapies for weight loss have been developed since the origin of DPP and are used for individuals without diabetes and with obesity. However, lifestyle modification and metformin remain the first-line therapies for prevention of type 2 diabetes (12), particularly in the absence of severe obesity, and this newly developed model aligns with current clinical practice.

In conclusion, this study provides the first type 2 diabetes risk prediction model with individualized preventive intervention effects based on data from a randomized controlled trial of metformin therapy or intensive lifestyle intervention. The race-free diabetes risk prediction model and equation were developed and validated in racially and ethnically diverse study populations. Parsimonious and commonly available clinical risk predictors coupled with the development of a free and publicly available online diabetes risk calculator enable high potential clinical utility of this research on individualized diabetes prevention. Further, this study provides the framework to develop risk prediction models with individualized preventive interventions for other chronic diseases and respective intervention strategies, potentially broadening the offering of precision preventive medicine.

## Data Availability

The DPP was conducted by the DPP investigators and supported by the National Institute of Diabetes and Digestive and Kidney Diseases (NIDDK). The data from the Diabetes Prevention Program (DPP) (DOI: 10.58020/3hw5-cf91) reported here were supplied by NIDDK Central Repository (NIDDK-CR) and are available for request at https://repository.niddk.nih.gov/studies/dpp/. This manuscript was not prepared under the auspices of the DPP study and does not necessarily reflect the opinions or views of the DPP study, NIDDK-CR, or NIDDK. Data for the Multi-Ethnic Study of Atherosclerosis were obtained via a data sharing agreement from the coordinating center and are publicly available https://biolincc.nhlbi.nih.gov/studies/mesa/.

## Prior Presentation

M.B. presented preliminary findings of this project at the American Diabetes Association Scientific Sessions as a poster presentation, June 21-24, 2024.
